# Editorial: Advancement of RWD/RWE utilization for enhancing drug development and benefit/risk assessment

**DOI:** 10.3389/fphar.2026.1787363

**Published:** 2026-01-28

**Authors:** Yoshiaki Uyama, Anick Bérard, K. Arnold Chan

**Affiliations:** 1 Center for Regulatory Science, Pharmaceuticals and Medical Devices Agency, Tokyo, Japan; 2 CHU Sainte Justine Research Center, University of Montreal, Montréal, QC, Canada; 3 TriNetX LLC., Cambridge, MA, United States

**Keywords:** database analysis, drug developement, pharmacoepidaemiology, real world evidence (RWE), risk/benefit assessment

Real-world data (RWD) and real-world evidence (RWE) have been utilized not only for drug safety assessment but also for effectiveness evaluation for drug approval on a global basis and many cases of RWD/RWE utilization for regulatory purposes have been accumulated with more diversifications in terms of data sources, methodology and study region (Kajiyama et al., 2024; Asano et al., 2025; Bachinger et al., 2025; Innes et al., 2025). Scientific discussions toward international harmonization related to RWD/RWE have also been more active within the International Council for Harmonisation of Technical Requirements for Pharmaceuticals for Human Use (ICH) such as ICH M14 and E23 (ICH, 2024; ICH, 2025).

The five studies presented under the recent Research Topic, Advancement of RWD/RWE Utilization for Enhancing Drug Development and Benefit/Risk Assessment illustrate both the progress achieved and the challenges that remain in the use of RWD for pharmacovigilance, regulatory science, and clinical decision-making. Each study addresses a distinct research question and highlight a central issue. Specifically, large-scale healthcare data were transformed into scientific evidence that is credible, interpretable, and fit for regulatory and clinical purposes.

The study by Suzuki et al. on Kampo medicines containing *Prunus persica* kernel exemplifies the constructive role of RWD in re-evaluating long-standing assumptions. Traditional contraindications during pregnancy have often been based on hypothetical concerns rather than empirical evidence. By applying pharmacoepidemiological methods to a large claims database and using an active comparator, the authors provide evidence suggesting no increased risk of preterm birth or major congenital malformations. This work demonstrates how RWD can complement traditional knowledge and support more balanced, evidence-based risk estimation, particularly in populations where randomized trials are rarely feasible.

However, the reliability of conclusions depends on the validity of study designs and variable definitions. The investigation into the use of validated claims-based algorithms in Japanese post-marketing database studies by Ishiguro and Nonaka highlights a persistent methodological weakness. Validated algorithms (also known as computable phenotype) were generally used for effectiveness issues but not safety issues, suggesting the need to promote the use of validated claims-based algorithms for enhancing the strength of evidence in pharmacoepidemiological studies. This gap underscores the need for stronger incentives, clearer expectations, and greater transparency regarding algorithm validity in real-world database studies.

The study by Yamazaki et al. further contextualizes the situation of regulatory-required post-marketing data sources in Japan. Most of database studies focused on important identified risks for mainly confirming incidence of adverse events in Japanese patients under real-world clinical settings, while important potential risks are less frequently addressed. It indicates an insufficient utilization of existing databases in Japan. Making full use of the advantage of database study such as large sample sizes, longitudinal follow-up, and availability of appropriate comparators by taking a more strategic approach could enhance early signal evaluation and reduce reliance on resource-intensive primary data Research Topic.

The methodological study comparing insurance-based and hospital-based databases in terms of outcome event coverage by Ando et al. suggests that the choice of data source directly affects the validity of outcome ascertainment and effect estimation. Under Japan’s universal healthcare system with universal access to medical institutions, substantial proportions of outcome events may occur outside of the hospital that the patient initially consulted. The demonstrated differences in outcome detections and their impact on effect estimates may have direct implications for both domestic studies and international collaborations. Explicit consideration of longitudinal capture of relevant events should therefore become standard practice in pharmacoepidemiological research.

Finally, the application of machine learning models to predict aminoglycoside-associated acute kidney injury by Zhang et al. points toward an evolving role for RWD from retrospective evaluation to prospective risk prediction. The strong predictive performance of advanced models such as XGBoost suggests potential clinical utility, although issues of transparency, reproducibility, and regulatory acceptability must be addressed before such models can be fully integrated into pharmacovigilance and regulatory practices.

In conclusion, this Research Topic reflects a field of RWD/RWE utilization in transition from merely using RWD to actively strengthening RWE for decision-making use. The five studies provide a multifaceted view of the current state and emerging challenges of RWD/RWE utilization in Japan and China. A common theme across the studies is the gap between data availability and the evidence generation that is sufficiently robust for regulatory and clinical decision-making. To further advance the field of RWD/RWE utilization for regulatory and clinical decision-making, it is important to accumulate more empirical and relevant examples utilizing RWD/RWE through active research in pharmacoepidemiology. Sharing knowledge and experiences including their limitations globally will be key to appropriately understanding the value of RWD/RWE and to creating a sustainable ecosystem for their utilization in drug development and benefit/risk assessment.
